# *SMART GROW* – Low-cost automated hydroponic system for urban farming

**DOI:** 10.1016/j.ohx.2023.e00498

**Published:** 2023-12-09

**Authors:** Kenny Kueh Yung Shin, Tan Ping Ping, Maybelline Goh Boon Ling, Chong Chee Jiun, Noor Alamshah B. Bolhassan

**Affiliations:** Faculty Computer Science and Information Technology, Pre-University, Universiti Malaysia Sarawak, 94300 Kota Samarahan, Sarawak, Malaysia

**Keywords:** Smart hydroponics, Android mobile application, ESP32, Urban agriculture

## Abstract

Urban farming has gained popularity in recent years, as more people have become interested in locally grown food and reducing their carbon footprint. Smart hydroponic systems can be an important tool for urban farming as they allow for precise control over plant growth and require less space and resources than traditional farming methods. Urban areas often lack access to land suitable for farming, making hydroponic systems a viable option for growing crops in limited space. Readily available hydroponic systems in the market are costly and not cost effective, thus hydroponic systems are usually only installed in larger scale farming. The challenge here is to connect multiple low-cost sensors to microcontrollers and to any store-bought hydroponic set. This paper describes the development of smart Internet of Things (IoT) hydroponic system integrated with an Android mobile application for small scale urban farming.

The new set up of IoT hydroponic set, coined as SMART GROW, is used to monitor and control various aspects of the system based on the basic parameters important in growing a healthy plant. The challenges faced during this build were irregular reading of the analog sensor when connected to a single board microcontroller (ESP32). This issue was resolved. SMART GROW currently is capable of monitoring basic parameters such as pH, EC and water level and can cater to additional sensors for monitoring other parameters if required. SMART GROW can easily be replicated and built at home and customized to the needs of the plant’s requirement. SMART GROW is versatile as it can be used to grow a wide variety of plants, including herbs, vegetables, and fruits, and offers several benefits over traditional soil-based growing methods such as automated regulation of the water level.

## Graphical abstract

**Figure:**
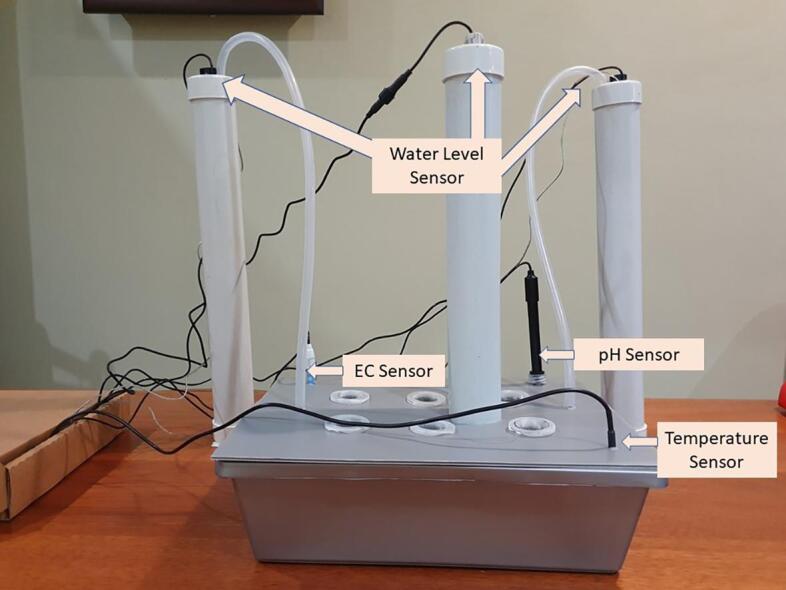

Specification Table

**Table:** 

Hardware name	Smart Home Hydroponics Kit
Subject area	• Environmental, planetary, and agricultural sciences • Open-source alternative to existing infrastructure
Hardware type	• Field measurements and sensors
Closest commercial analog	*“No commercial analog is available.”*
Open-source license	GNU General Public License (GPL)*.*
Cost of hardware	*RM 314.48 (∼72 USD)*
Source file repository	https://data.mendeley.com/datasets/t4n3t4p6db/4

## Hardware in context

Urban agriculture is a growing trend that includes various techniques such as vertical farming, indoor farming, hydroponics, aeroponics, aquaculture, and aquaponics [1]. Hydroponics is a form of vertical farming that grows plants in nutrient solutions instead of soil [2]. It can be done with or without the use of smart technology. Smart hydroponic systems are designed to improve water use efficiency by applying a combination of hydroponic systems with drip irrigation [3].

Recent studies have shown that integrating wireless sensor networks and the Internet of Things (IoT) into hydroponic systems can assist in optimal plant growth [1]. The architectural design of smart hydroponics systems has evolved over the past ten years or more [4], [5], from purely hardware components [6], [7] to designs that include server and client services [5], [8], [9]. The ability of smart hydroponics has evolved from monitoring only to providing corrective ability to maintain equilibrium required by the hydroponics system [10], [11], [12].

Smart hydroponics systems commonly consist of the following components:

1. Hydroponics hardware that provides a planting platform,
2. Data acquisition hardware to capture parameters of the hydroponic solution,
3. Server processing that analyses data captured by the data acquisition, and
4. Client services that provide different choices of services at the user end.

Remote monitoring and automatic regulation of hydroponic systems have been achieved with systems consisting of a network of devices allowing the value of sensors to be remotely monitored and controlled in real-time. These systems feature the ability to self-regulate with minimal user intervention. For example, [7] developed an auto-calibrated pH sensor capable of detecting and correcting out-of-range pH readings through an attached irrigation system. However, users could not remotely identify the pH reading in real-time.

The choice of hydroponics hardware depends on regional needs, with most literature choosing nutrient film techniques as less water is required [4]. Sensors deployed in the data acquisition system mostly focus on the water level and pH reading of the system [13]. More recent designs have deployed more sensors to detect other parameters such as EC reading and temperature of water [9]. The processor connecting directly to the sensors at the hardware level has shifted from Arduino UNO [8] to Arduino MEGA boards [14], and later to custom-made PCB boards or ESP32 boards [9], [15] that have higher clock speeds and WiFi features in their circuitry.

For data transfer protocols, WiFi and LoRaWAN are more commonly chosen [5]. The server processing has Thingspeak as the most frequently found service deployed by the literatures. These literatures do not provide client service in the form of a mobile application interface because their main purpose is to minimize user manual intervention. The client service is normally in the form of notification and does not receive input from the user [5], [13]. Notifications are only sent to the user when critical situations occur, such as when water levels are lower than normal [5]. [8] uses Firebase, which allows connection to a client’s mobile application, but it did not report any GUI. However, there are situations where user input is required. Our study proposes a system that allows customizing parameters of system to suit different plants, which is not addressed in most literatures. Although [14] reported trials for different vegetables planted in their system, they did not explain in detail how the changes to parameters were made.

In addition, the system proposed by [14] costs around 400 USD, which is relatively high. According to [16], the cost of a small, uncomplicated hydroponic system typically ranges from USD 50 to USD 500. Medium systems cost at least several hundred dollars, while large systems can cost several thousand dollars or more. In developing countries like Malaysia, a ready-to-use 4-tier waterfall home garden costs RM 599 (USD 130) [17], while a DIY hydroponic set with a timer costs RM 229 (USD 50) [18], both without IoT. A ready-to-use 32-pot full-spectrum system including a timer, pH meter, and digital timer but without mobile app IoT control costs RM 1099 (USD 240) [19]. SMART GROW outperforms these systems at a lower cost, making it more affordable and customizable for developing countries.

While there are existing works on automated hydroponic systems [10], [11], [12], no specific steps or costs are given. Four commercial automated smart hydroponic systems were reviewed for their cost, features, graphical user interface design, and pros and cons. Additionally, the tools and techniques used to develop the prototype were examined for their suitability for this project. Table 1 summarizes the comparisons made.

**Table 1:** Comparison of features to Existing Commercial Systems.

	System			
Features	Existing System			Proposed System
	LEAF [10]	Grobo One [11]	Seedo Home [12]	SMART GROW
Connect to Wi-Fi	Yes	Yes	Yes	Yes
Monitor hydroponic system in real-time	Yes	Yes	Yes	Yes
Regulate hydroponics’ environment automatically	Yes	Yes	Yes	Yes
Notify user through mobile app when system faulty	No	No	Yes	Yes
Number of Plant	1	1	2	6
Supported Platform	Android/iOS	Web	Android/iOS	Android
Cost	2690 USD	1999 USD	2400 USD	∼ 72 USD

## Hardware description

**Fig. 1:**
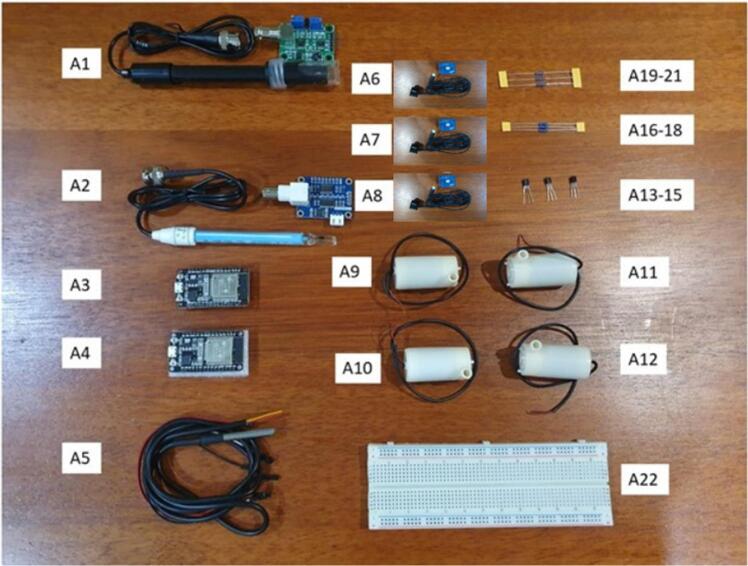
Image of Hardware Components of the SMART GROW Systems.

From the requirement analysis which captures an unambiguous and complete picture of the proposed smart hydroponic system, SMART GROW consists of a DIY Hydroponics Set with 6 Holes, sensors for pH, EC and ultrasonic to monitor the water level and ESP32 microcontroller to link the sensors to WIFI. The full list of hardware is shown in Table 2 and Fig. 1 shows the image of the hardware components.

**Table 2:** Overview of Parts and Description of their Function.

Label	Part Name	Description
A1	pH circuit board and pH Probe	Detect pH level of the solution in the hydroponic reservoir
A2	EC circuit board and EC Probe	Detect EC level of the solution in the hydroponic reservoir
A3	ESP32 Microcontroller	Send and receive data from EC sensor to Firebase
A4	ESP32 Microcontroller	Manage all module except EC sensor including send and receive data from sensors to Firebase and environment regulation
A5	Temperature Sensor	Detect temperature of solution in the hydroponic tank
A6	Ultrasonic Sensor 1	Detect the water level in the hydroponic tank
A7	Ultrasonic Sensor 2	Detect the water level in the clean water tank
A8	Ultrasonic Sensor 3	Detect the water level in the nutrient solution tank
A9	Water Pump 1	Pump water out of hydroponic tank if the water level is too high
A10	Water Pump 2	Pump clean water into hydroponic tank if the pH or EC value is too high
A11	Water Pump 3	Pump nutrient solution into hydroponic tank if the pH or EC value is too low
A12	Water Pump 4	Pump that circulates the flow of solution inside the hydroponic tank
A13	Transistor BC639 1	To act as switch for Water Pump 2
A14	Transistor BC639 2	To act as switch for Water Pump 3
A15	Transistor BC639 3	To act as switch for Water Pump 1
A16	Resistor 220 Ohm 1	To act as base resistor for Transistor 1
A17	Resistor 220 Ohm 2	To act as base resistor for Transistor 2
A18	Resistor 220 Ohm 3	To act as base resistor for Transistor 3
A19	Resistor 1000 Ohm 1	To act as voltage divider for EC Sensor
A20	Resistor 1000 Ohm 2	To act as voltage divider for pH Sensor
A21	Resistor 1000 Ohm 3	To act as voltage divider for Temperature Sensor
A22	MB102 Bread Board	For wiring electrical circuits

### Espressif ESP32 development board

The ESP32 is a microcontroller that can readily connect to the Internet via Wi-Fi. The SMART GROW system utilizes two DOIT ESP32 DEVKIT V1 boards. One board is used to capture and upload data from the EC probe. The second board is used to capture and upload data from the pH probe and ultrasonic sensors, fetch data from the database, including ECValue, and control micro water pumps 1, 2, and 3.

### PH4502C pH sensor

The pH sensor is connected to one of the ESP32 boards. However, the pH sensor outputs an analog signal ranging from 0 to 5 V, while the ESP32 can only receive voltage inputs of 3.3 V or below, creating an incompatibility. This issue was resolved by using two 1000 Ohm resistors to lower the voltage to below 3.3 V before it reaches the ESP32. The output was calibrated through calculations using polynomial regression at different points. The regression equation was proposed by an engineer through empirical processes and integrated into the SMART GROW Arduino codes. User can directly use the code without further calibration. The pH sensor serves as a pH level detector, with codes written to continuously fetch data from the sensor and send it to the Firebase database via the ESP32.

### DFR0300 EC sensor

The EC sensor is connected to a different ESP32 board than the pH sensor. Similar to the pH sensor, the EC sensor outputs an analog signal greater than 3.3 V, while the ESP32 can only receive voltage inputs of 3.3 V or below, creating an incompatibility. This issue was resolved by using a single 1000 Ohm resistor to lower the maximum output of the EC sensor. The output was calibrated through the same calculations that uses polynomial regression at different points which was proposed by an engineer. The EC sensor must be connected to a separate ESP32 board to avoid electrical interference with the pH sensor and achieve stable readings.

### DS18B20 temperature sensor

The temperature sensor is connected to the same ESP32 board as the EC sensor. This is necessary because the temperature of the hydroponic solution is required for the computation of the EC value, which is integrated into the SMART GROW Arduino codes. Users can directly use the code without further configuration. The waterproof temperature sensor is placed in the hydroponic solution to measure its temperature.

### JSN-SR04T ultrasonic sensor

Three ultrasonic sensors are utilized in this project, each serving a distinct function. The first sensor measures the height of the water level in the hydroponic reservoir, the second sensor measures the height of clean water in the water reservoir, and the third sensor measures the height of the nutrient solution in the nutrient reservoir. All readings are calculated and converted into percentages to facilitate user understanding.

### Micro water pump

Four micro water pumps are utilized in this project, each serving a distinct function. The first pump is responsible for pumping clean water into the hydroponic reservoir if the pH or EC value exceeds the user-defined threshold. The second pump adds nutrient solution to the hydroponic tank when the pH or EC value falls below the user-defined threshold. The third pump removes excess water from the hydroponic reservoir, while the fourth pump circulates nutrient-enriched water through the hydroponic tiers.

### Software overview

The Arduino Integrated Development Environment (IDE) is an open-source software that allows users to write, compile, and upload code to development boards. It is available for free on multiple platforms, including Windows, macOS, and Linux. The IDE is designed to be user-friendly and accessible to beginners while also being powerful enough for experienced programmers. It includes a code editor, compiler, debugger, and serial monitor for communicating with the board and monitoring its output.

One advantage of using the Arduino IDE when programming ESP32 microcontrollers is its compatibility with the ESP32-Arduino Core software. This software bridges the hardware gap between the ESP32 and the Arduino, allowing users to use the Arduino IDE as their development environment and program the ESP32 using a language similar to that used for the Arduino. Additionally, many Arduino libraries can be reused when writing software for the ESP32.

Users can write code in C++ using the Arduino IDE and upload it to development boards. The IDE includes a large library of pre-written code and examples that can be used to learn how to program different types of development boards. For instance, SMART GROW was coded using C Programming Language as Arduino IDE supports both C and C++.

## Design files summary

All packages and files can be downloaded from the Mendeley repository, with links provided in Table 3.

**Table 3:** Design Files Summary.

Design File Name	File Type	Open-Source License	Location of File
Hardware Assemble - Schematic Diagram	Image file (pdf)	GNU General Public License	https://data.mendeley.com/datasets/t4n3t4p6db/4
Blueprint of DIY SMART GROW Hydroponic Set	Image file (pdf)	GNU General Public License	https://data.mendeley.com/datasets/t4n3t4p6db/4
Arduino Libraries	Arduino Libraries	GNU General Public License	https://data.mendeley.com/datasets/t4n3t4p6db/4
Hardware Program - Arduino Coding	Arduino Code (ino)	GNU General Public License	https://data.mendeley.com/datasets/t4n3t4p6db/4
Database Diagram	Image file (jpeg)	GNU General Public License	https://data.mendeley.com/datasets/t4n3t4p6db/4
Mobile application	Android Package Kit (apk)	GNU General Public License	https://data.mendeley.com/datasets/t4n3t4p6db/4

## Bill of materials summary

**Table:** 

Designator	Component	Number	Cost per unit (RM)	Total cost* (RM)	Source of materials	Material type
A1	pH Sensor	1	47.88	47.88	https://www.lazada.com.my/	Electronic
A2	EC Sensor	1	68.00	68.00	https://www.lazada.com.my/	Electronic
A3-A4	ESP32 boards	2	25.00	50.00	https://www.lazada.com.my/	Electronic
A5	Temperature Sensor	1	10.00	10.00	https://www.lazada.com.my	Electronic
A6 – A8	Ultrasonic Sensor	3	17.59	53.85	https://www.lazada.com.my/	Electronic
A9 – A12	Micro Water Pumps	4	12.20	48.80	https://www.lazada.com.my/	Electronic
A13-A15	Transistors	3	0.35	1.05	https://www.lazada.com.my/	Electronic
A16-A21	Resistors	6	0.10	0.60	https://www.lazada.com.my/	Electronic
A22	Bread Board	1	5.00	5.00	https://www.lazada.com.my/	Electronic
Hydroponic Set	Plastic Tray	1	2.30	2.30	Store bought	PVC
Hydroponic Set	Impra Board 680 mm × 766 mm	13	8.003.00	8.00 9.00	Store bought Store bought	Plastic PVC
Nutrient/Water/Hydroponic Reservoir Tower	PVC Pipe					
Nutrient/Water/Hydroponic Reservoir Tower	PVC Cap	5	2.00	10.00	Store bought	PVC

			Total	314.48

• *Include delivery fees.*

## Build instructions

### Overall SMART GROW system

**Fig. 2:**
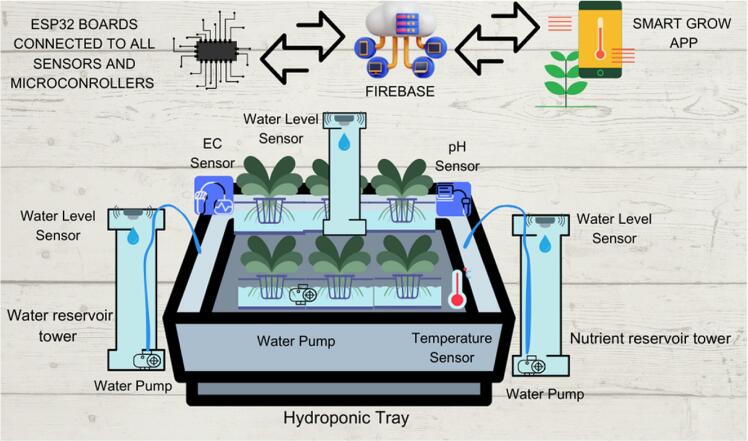
Illustration of the Overall Hydroponic Set.

Fig. 2 depicts the overall set up of SMART GROW. SMART GROW is hydroponics with IoT (Internet of Things) which will enhance the efficiency and productivity of hydroponic systems. Hydroponics itself is a method of growing plants without soil, where the plants are instead grown in a nutrient-rich water solution. The illustration below shows the entire SMART GROW set up which consists of the DIY 6 holes hydroponic set, sensors linked to the ESP32 boards, firebase and SMART GROW application.

### DIY SMART GROW hydroponic set

**Fig. 3:**
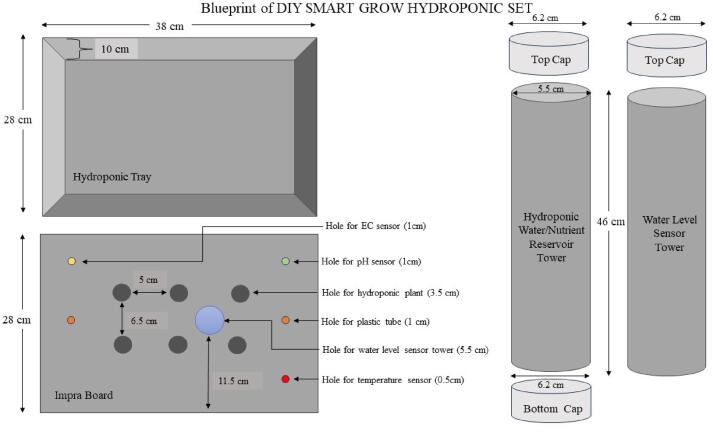
Blueprint of SMART GROW DIY Hydroponic Set.

The hydroponic set was customized and built from scratch to minimize cost. Fig. 3 is the blueprint of SMART GROW DIY Hydroponic set. The maximum capacity of SMART GROW is 10 L. The inner dimensions of the tray were 38 cm (L) × 28 cm (W) × 10 cm (H), which would give a volume equal to 10,640 cm^3^. The container was filed with a 10 L of water and the water needed to reach a certain height depending on the container dimension, which was calculated using the equation below:

$$\mathrm{WaterHeadHeight}=\frac{\mathrm{LiterofWater}\times 1000}{L\times W}$$

Therefore, for 10 L of water, the equivalent height of the water inside the container will be almost equal to 9.40 cm.

**Fig. 4:**
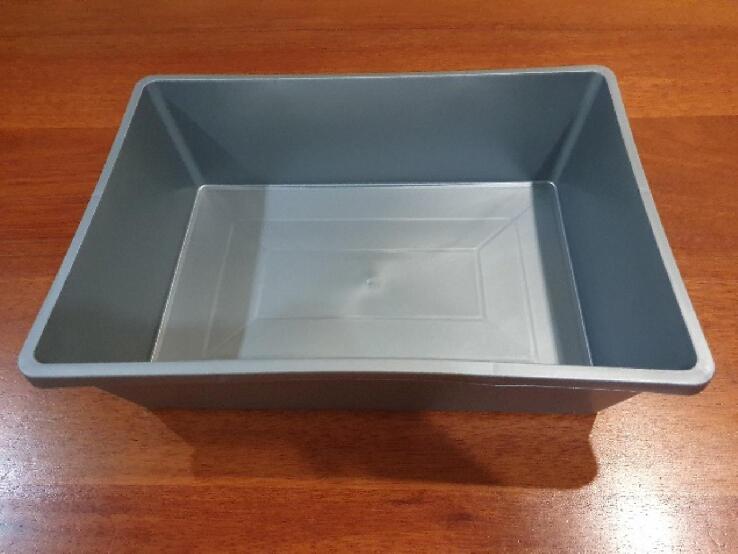
Plastic Tray.

**Fig. 5:**
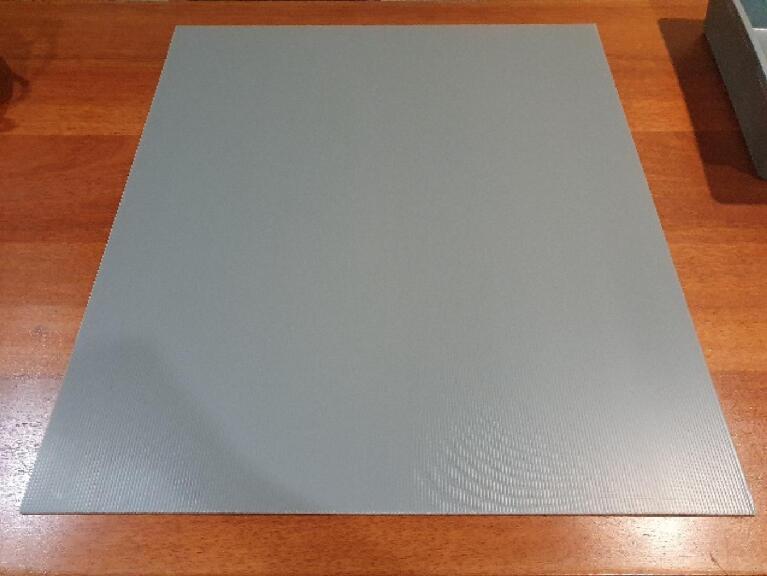
Impra Board.

**Fig. 6:**
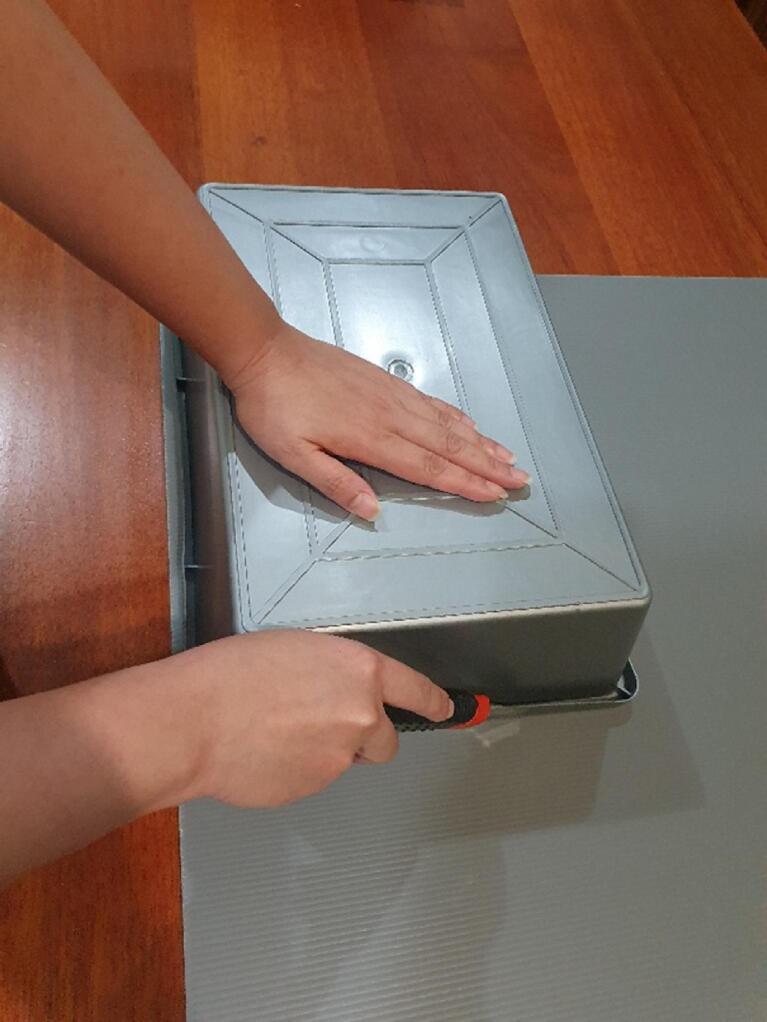
Showing the Impra board being cut to size.

**Fig. 7:**
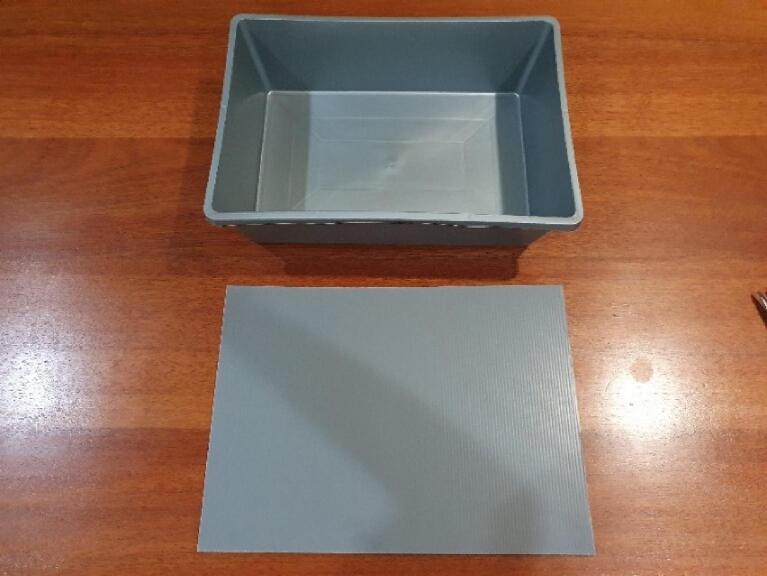
Showing the Impra board already cut to size.

**Fig. 8:**
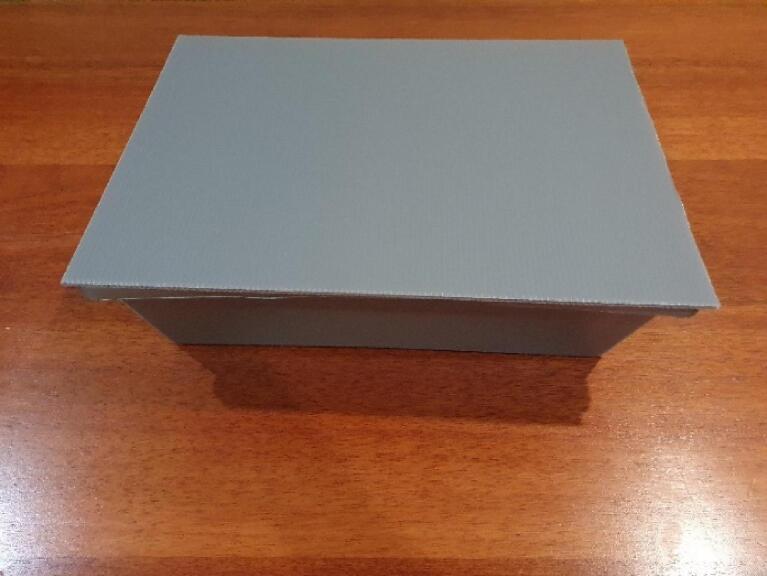
Showing the Hydroponic Box.

Fig. 4 until Fig. 8 show the actual items used to build the DIY SMART GROW hydroponic set (see Fig. 5, Fig. 6, Fig. 7).

**Fig. 9:**
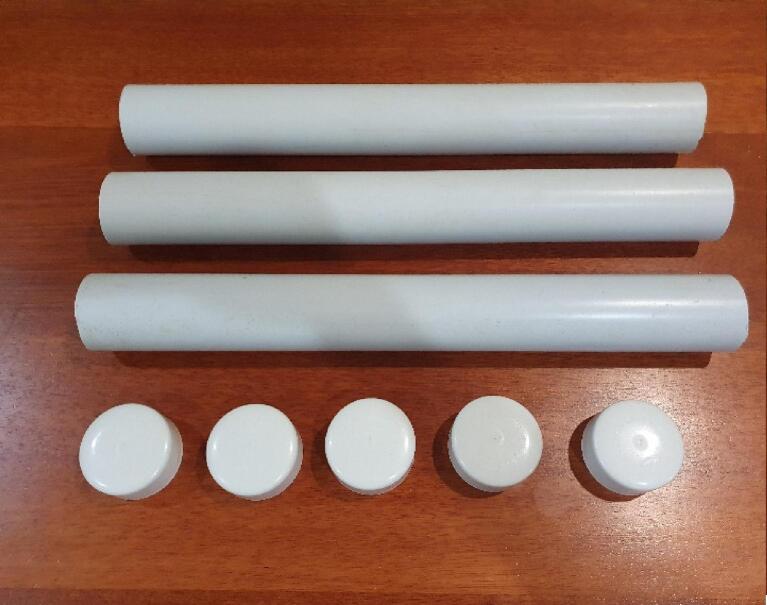
Showing the PVC pipe and cap.

**Fig. 10:**
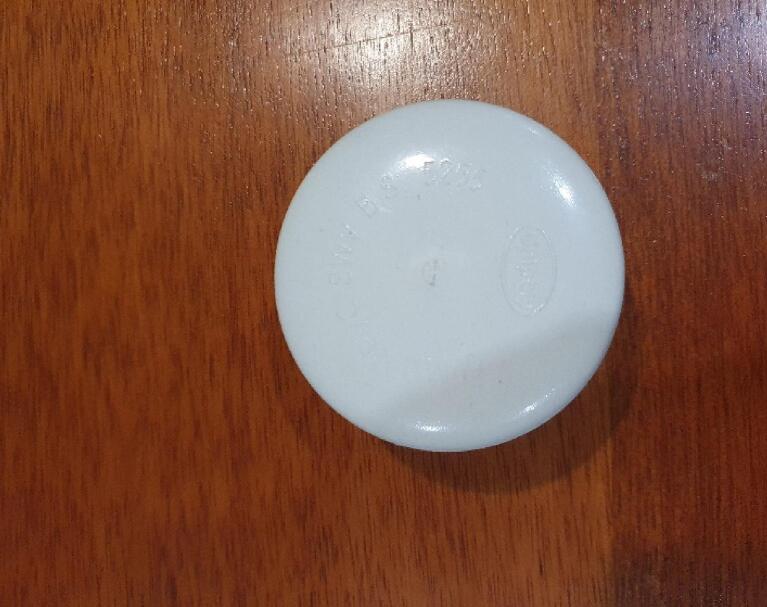
Showing cap used (BS 5255).

**Fig. 11:**
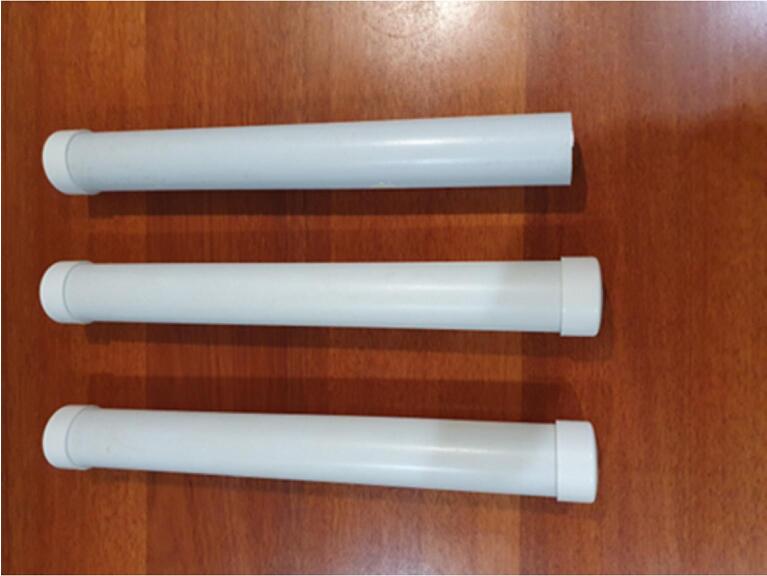
Completed Hydroponic Reservoir/Sensor Tower after assembly.

Fig. 9, Fig. 10, Fig. 11 shows the components for building nutrient/ water reservoir tower and water level sensor tower.

### Instruction to connect the sensors to the Arduino board

**Fig. 12:**
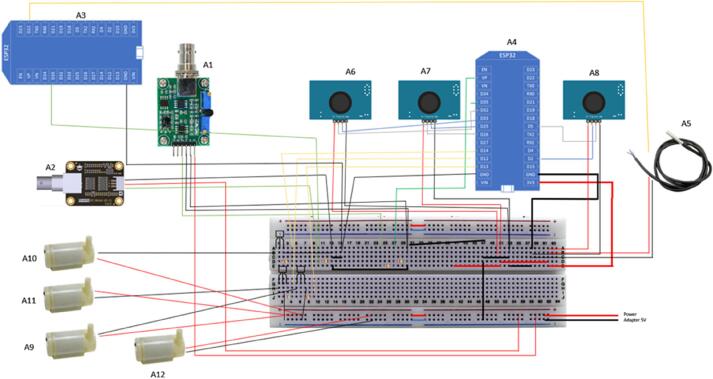
Pictorial Circuit Diagram of the Electronics Component of SMART GROW System.

**Fig. 13:**
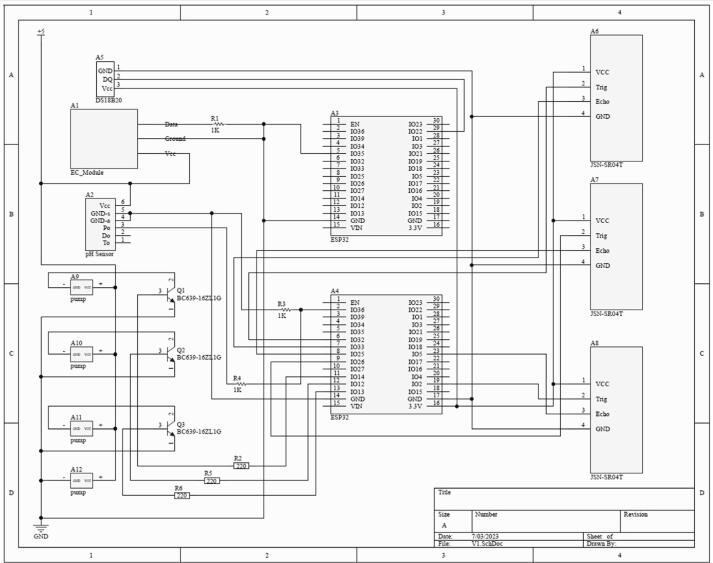
Schematic Circuit Diagram of the Electronics Components of SMART GROW System.

**Fig. 14:**
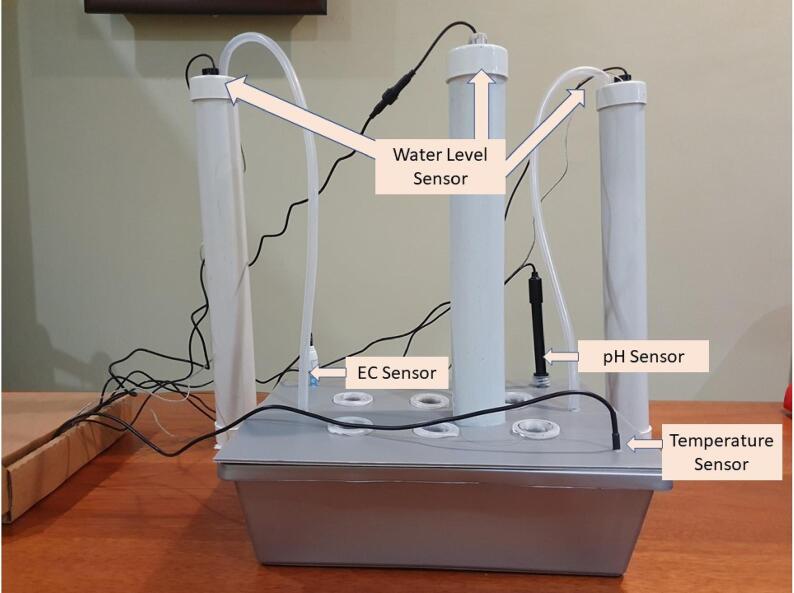
Completed Hydroponic Set Up.

Fig. 12 shows the pictorial circuit diagram that is equivalent to the schematic circuit diagram shown in Fig. 13. During the installation, user must make sure the power supply is not turned on. Table 2 provides a detailed overview of each hardware component and its functionality. Note that most of these components are not waterproof. Installation of the components must be done in dry area. Fig. 14 shows the complete setup of the hydroponic set with sensors.

### Instructions for programming the ESP32 board

First, the user must download and install the Arduino Integrated Development Environment (IDE). Then, they must install the ESP32 library by choosing version 1.0.6. A step-by-step guide can be viewed in the following video tutorial: https://www.youtube.com/watch?v=mBaS3YnqDaU. After that, the user must download the SMART GROW scripts and libraries from the link provided in this article (see Section 3). The SMART GROW libraries must then be downloaded and stored at the path for the Arduino IDE library (for example, on a desktop with Windows OS, the path would be …\Arduino\libraries). Next, the user must connect the ESP32 to a computer using a USB cable and open the Arduino IDE on the computer. The script should then be verified and uploaded to the ESP32. Note that there are two ESP32 boards deployed in the system. User must upload one of the codes to one of the ESP32s while uploading the other code to the other ESP32.

### Instructions for setting up a firebase real-time database

Download the SMART GROW database diagram (See Section 3). Create a Google Firebase Real-Time database account and create tables in it according to the database diagram.

### Instructions for installing the SMART GROW mobile application

First, user must download the APK file (see Section 3) to their devices. Before installing it, user must ensure that their devices are set up to allow the installation of apps from unknown sources. To do this, navigate to the device’s Settings, then to Security, and enable the “Unknown sources” option. Once the APK file has been downloaded and the “Unknown sources” option has been enabled, user can navigate to the folder where it is saved using a file manager app. Tap on it to start the installation process. User will be prompted to review the app’s permissions and then asked to confirm that they want to install it. After confirming, it will be installed on their devices and can be found in their app drawer or on their home screen, depending on their device.

### Operating instructions for SMART GROW Android mobile app

**Fig. 15:**
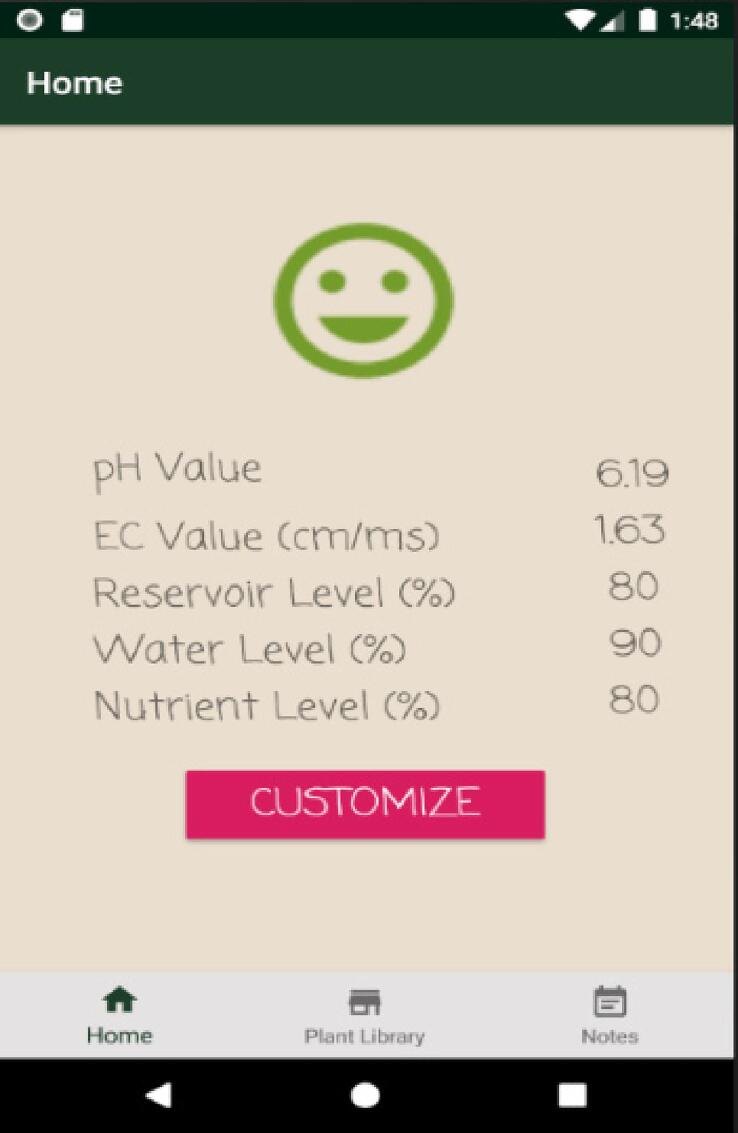
SMART GROW Homepage.

**Fig. 16:**
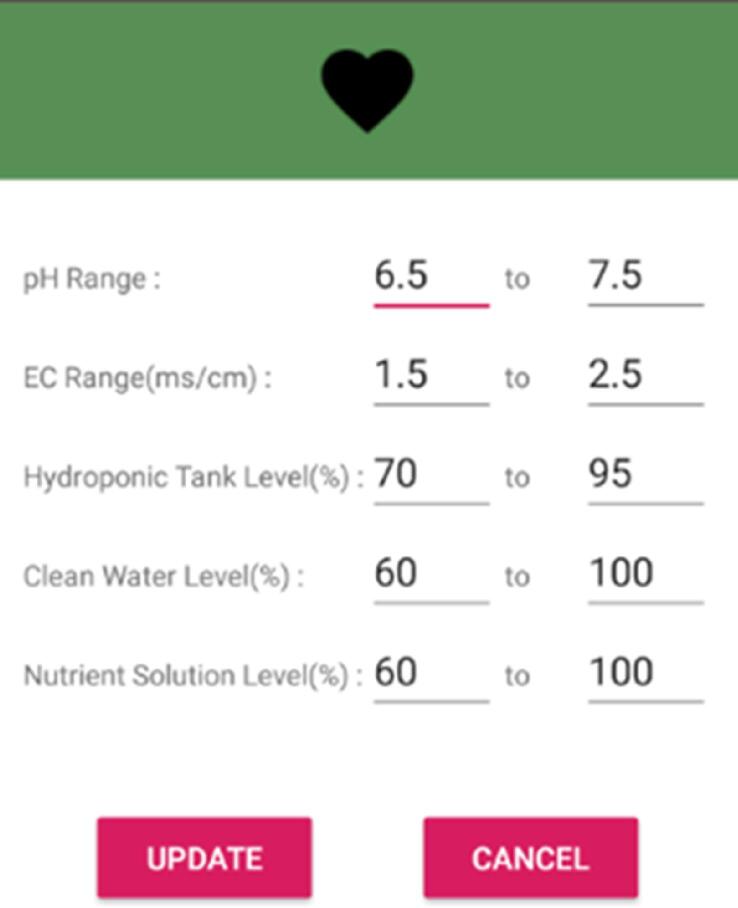
Edit current range dialog.

From the SMART GROW Android mobile app’s homepage, current readings of the hydroponic set are displayed (Fig. 15). A smiley face indicates that conditions are within range. This user interface is updated from time to time once data has been uploaded to the database by the hydroponic system. User can customize their desired range and it will regulate itself according to their settings (Fig. 16).

**Fig. 17:**
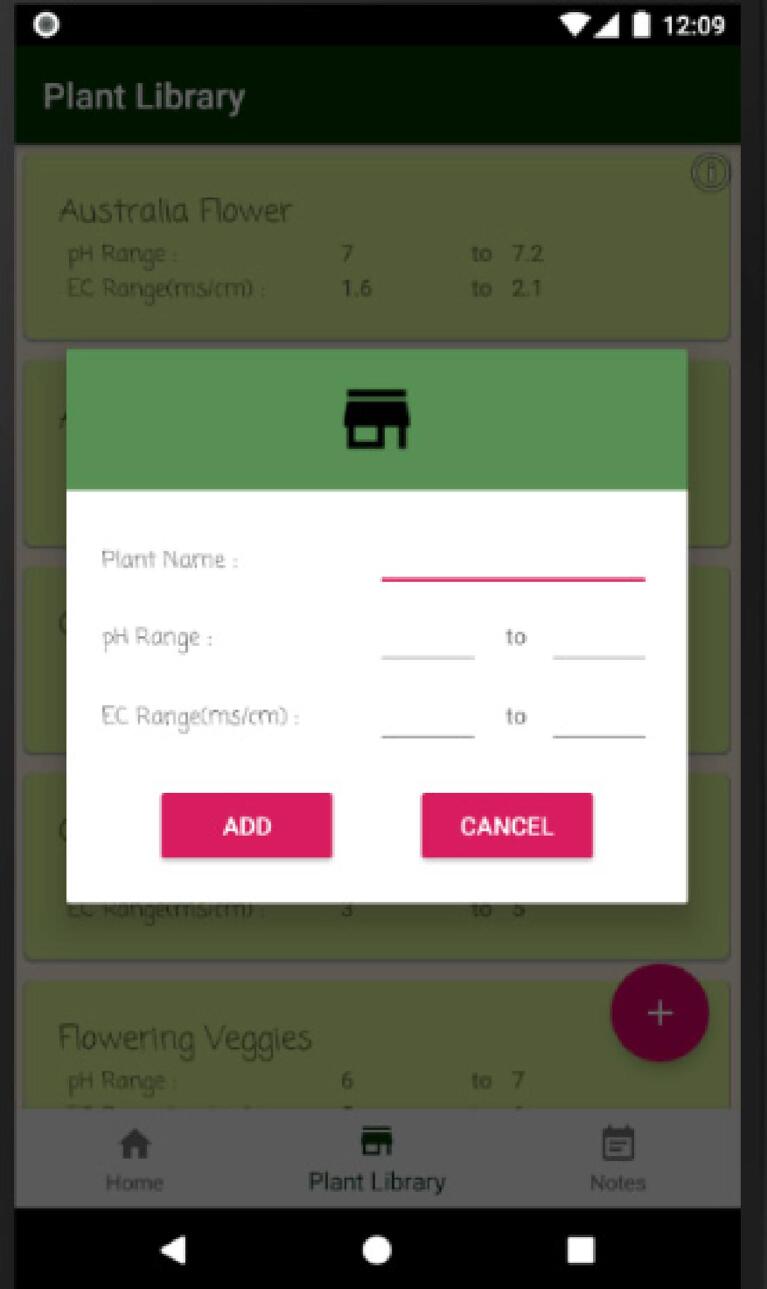
Add new plant dialog 1.

User can add new plant information (Fig. 17), which can be selected to regulate hydroponic environment conditions appropriately. They can also update or delete an existing plant information in the plant library tab.

**Fig. 18:**
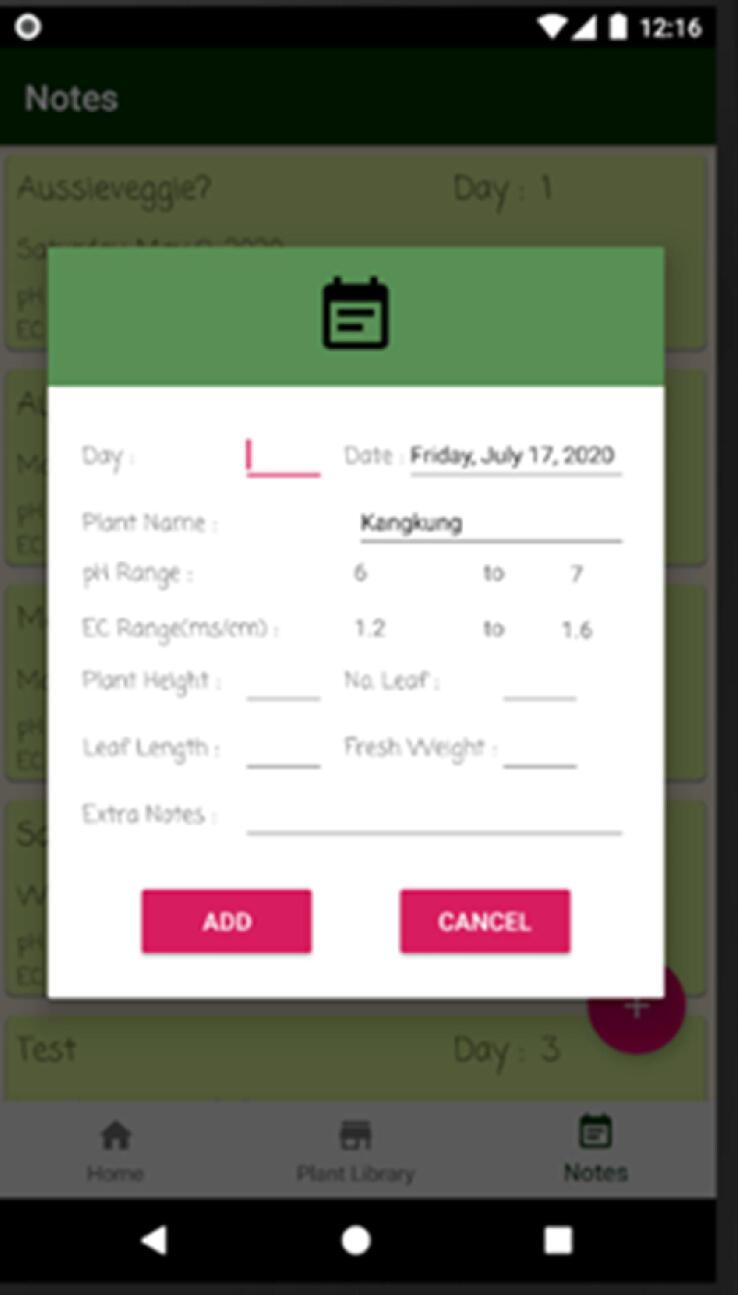
Add new plant dialog 2.

Lastly, user can add new notes (Fig. 18) reflecting plant information on a particular day by accessing this feature through an add new note dialog. Notes can also be updated or deleted in the note tab.

## Operation instructions

After completing the hardware setup as described in Section 5, the system operates according to the process shown in Figs. 19a, 19b, 20a, and 20b. The processes outlined in blue boxes must be completed by the user, while those in the remaining boxes are executed automatically by both ESP32 units. It is important to ensure that the host and authentication ID for the Firebase database are configured to the user’s own account.

**Fig. 19a:**
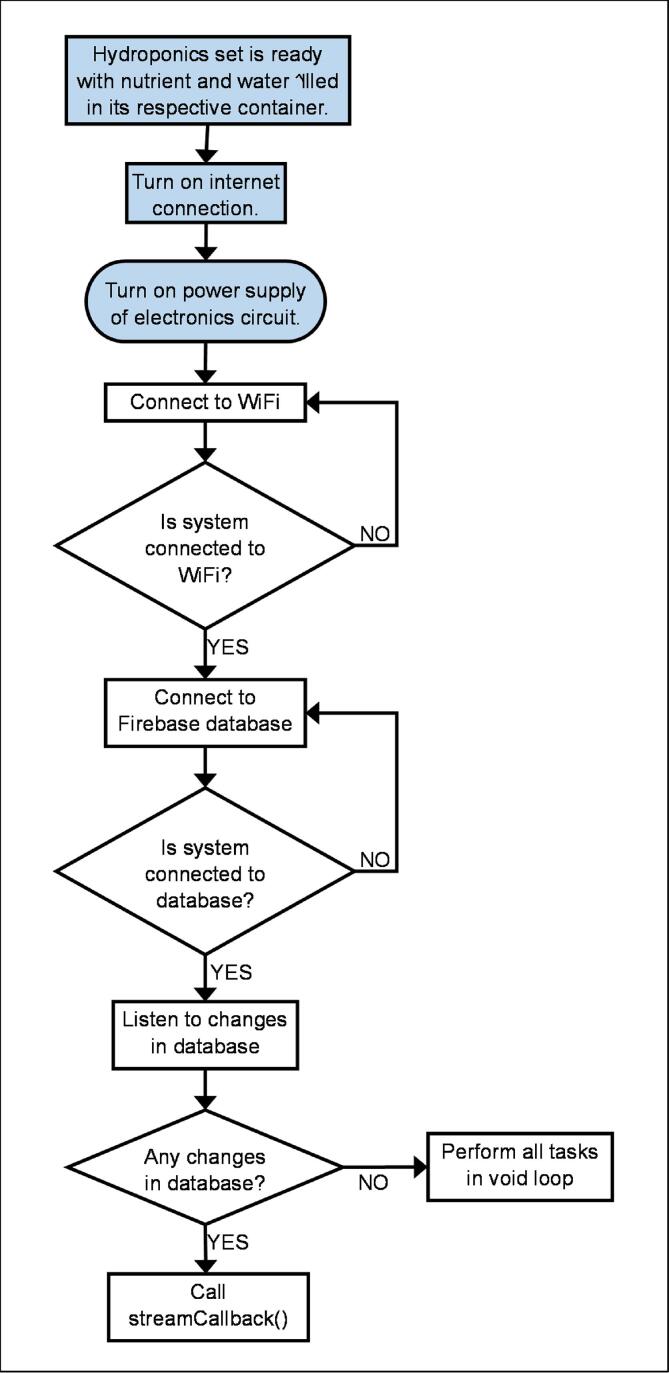
Overall Processes Occurring in ESP32 A4.

**Fig. 19b:**
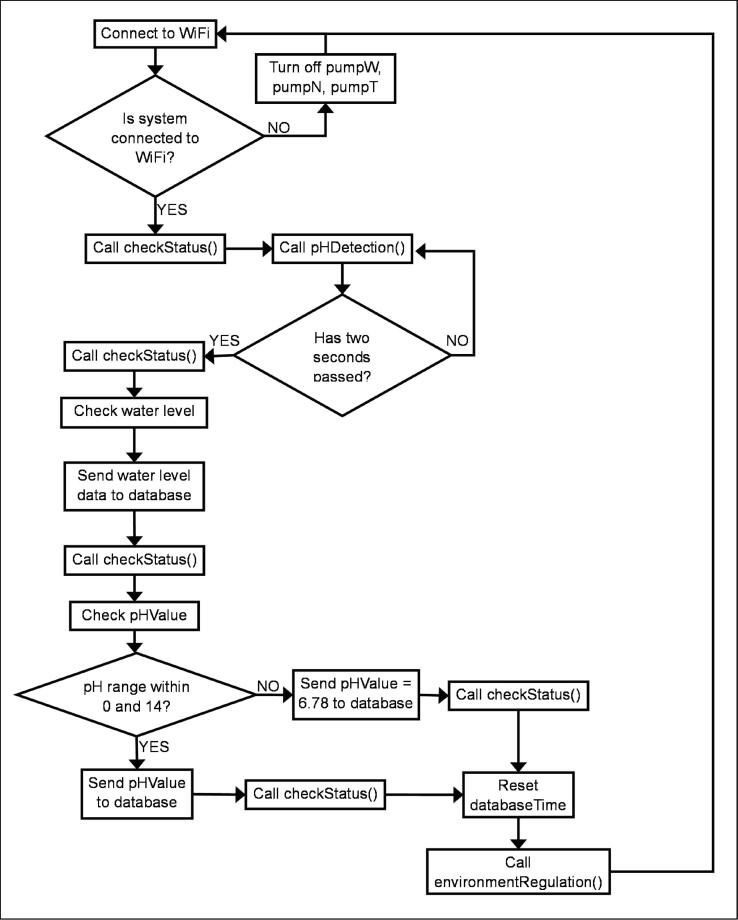
Automated Processes Repeatedly Carried Out Over Time as Long as the Power Supply to ESP32 A4 is Turned On.

**Fig. 20a:**
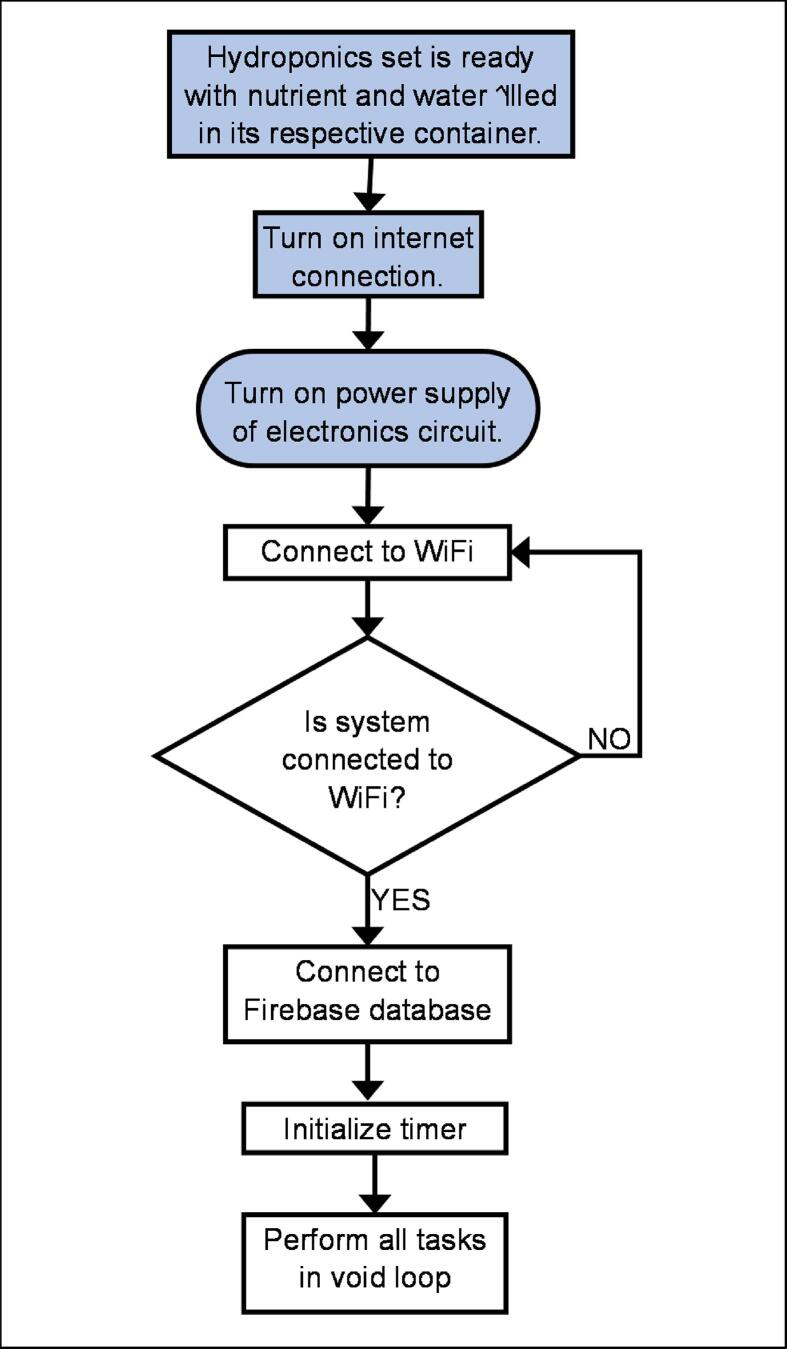
Overall Processes Occurring in ESP32 A3.

**Fig. 20b:**
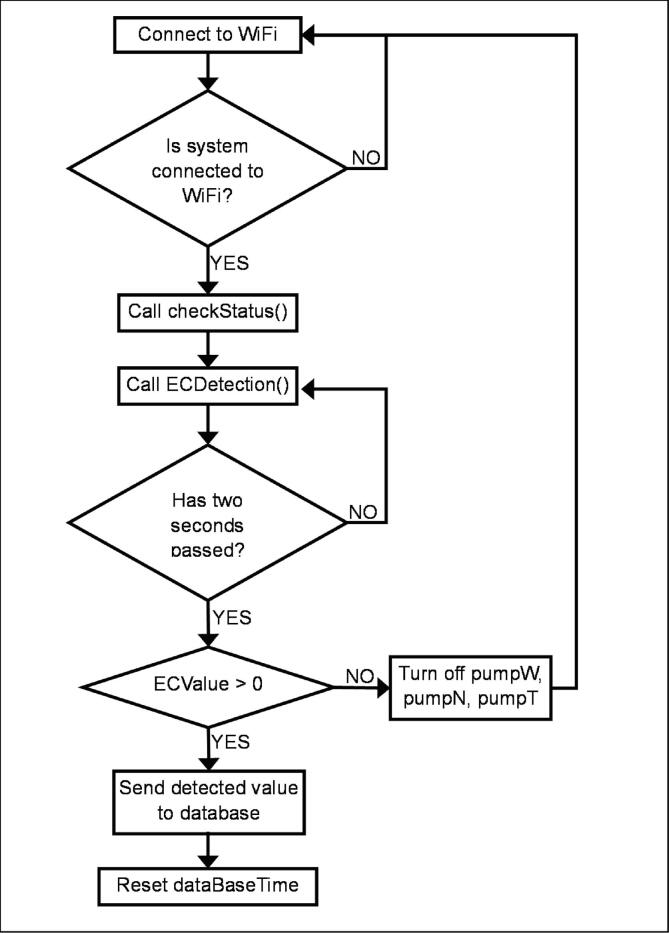
Automated Processes Repeatedly Carried Out Over Time as Long as the Power Supply to ESP32 A3 is Turned On.

In general, ESP32 A4 captures data from the pH probe and ultrasonic sensors and sends it to the database. It also controls all the micro water pumps and is responsible for streaming data from the database and updating the variables minrpH, maxrpH, minrEC, maxrEC, minrTank, and maxrTank locally. This only occurs when the Firebase library detects changes in data in the database. When environmentRegulation() is called, the state of three pumps - pumpW, pumpN, and pumpT - is either turned on or off based on the latest ECValue, pHValue and air level in the reservoir. PumpW supplies additional water to the hydroponics if it lacks water; pumpN supplies additional nutrients to the system if it lacks nutrients; and pumpT supplies additional water to the water container containing pumpW if its water level becomes too low to support the system. On the other hand, ESP32 A3 only captures data from the EC probe and sends it to the database regularly over time.

## Validation and characterization

The proof-of-concept is when the hydroponic set, IoT and the app must work together and ensure that the requirements of the users are achieved. The proof-of-concept for SMART GROW focuses on integration testing, functional testing, and user testing of the proposed system. There are two parts to the integration testing. The first part tests the operability of the low-cost sensors integrated on a single microcontroller. The second part was the integration testing, where all the sensors in the SMART GROW must be able to relay accurate data to the app developed. Each of the data is explained in the separate sub section 7.1.

This was then followed by functional testing which was to ensure that the developed app is in accordance with the functions required by the user. Sub section 7.2 will explain the results further.

Integration and functional testing are insufficient without the user utilizing and growing with the proposed system. Testing was conducted with user growing vegetables using the SMART GROW hydroponic system and the test results are explained in sub section 7.3 and 7.4.

### Integration testing

EC sensor, ultrasonic sensors and ESP32 were integrated and tested for the purpose of measuring electrical conductivity and water level. The first test carried out was not successful due to the placement of the EC sensor and the ultrasonic sensors on the same ESP32. The solution to this is to separate the EC sensor and ultrasonic sensors on two different ESP32 with two different circuits which when tested, showed accurate readings. This was an important setup in SMART GROW.

The pH and ultrasonic sensors were integrated with the ESP32 and tested for their ability to measure pH, nutrient, and solution levels in the tower. Preliminary tests were unsuccessful due to the placement of the sensors. The ultrasonic sensors require a clearance of 35 cm from the solution surface to accurately read the solution level. To resolve this issue, the pH sensors were placed further away, and the ultrasonic sensors were placed on top of the tower. Additionally, the pH and ultrasonic sensors were connected to different GND pins on the ESP32 to avoid ground loops, which can cause interference from currents generated by the sensors. With these adjustments, the pH readings fluctuated within an acceptable error margin, and the ultrasonic sensors were able to accurately read the water level.

The sensors and ESP32 are integrated as part of the SMART GROW and the purpose of the following integration testing was to assess the communication between the SMART GROW and the app. For this testing, the settings were changed in the SMART GROW app, when manually evaluated, these settings were applied to the SMART GROW box: providing a pass to this integration testing.

### Functional testing

In order to perform basic hydroponic plant monitoring, nine functions were requested and listed in table below (Table 4). Functional testing was performed based on each of these functions. All of the functions passed the functional testing because the functions was developed in accordance to the requirement listed by users.

**Table 4:** List of Functional Requirements of the SMART GROW App.

SMART GROW Application Function
View Current Condition of Hydroponic Environment
Update Current Requirement
Add Plant into Library
Select Plant
Update Plant Requirement
Delete Plant from Library
Add Plant Note into Library
Update Plant Note in Library
Delete Note from Library

### User testing

A user test was conducted to evaluate the proposed SMART GROW system. The Technology Acceptance Model (TAM) [17], a theory that models how users accept and use technology, was applied to the user testing of the hydroponic system. Two main aspects were considered:

• *Perceived usefulness* or how users find the system useful for a specific task.
• *Perceived ease-of-use* or how easily users can handle the system with minimal guidance.

The following factors were considered during user testing:

• Learn ability and intuitiveness.
• Error prevention and error handling
• Visibility and documentation
• Discoverability and exploration
• Consistency
• Interactivity

Users were provided with the SMART GROW system, including both the physical setup and the app, as well as a user testing form with the above factors listed for rating. Based on the user testing results, three factors - learnability and intuitiveness, error prevention and error handling, and consistency - passed. However, visibility and documentation, discoverability and exploration, and interactivity were rated as minor failures.

For visibility and documentation, users initially found it difficult to update or delete available plant data. However, this issue was quickly resolved through exploration of the available options. Although users rated discoverability and exploration as a minor failure, they did not test all of the functions available in the SMART GROW app within the given time frame. Nevertheless, they were able to view the main features. This issue was addressed by allowing users more time for exploration.

For interactivity, developers expected that users would use the app occasionally throughout the day, compared to current practice of manually checking the hydroponic set only when remembered. In the user testing form, users were asked if they used the app occasionally; they reported using it once or twice per day, which was better than expected. Overall, users were satisfied with the proposed system.

### User planting

**Fig. 21:**
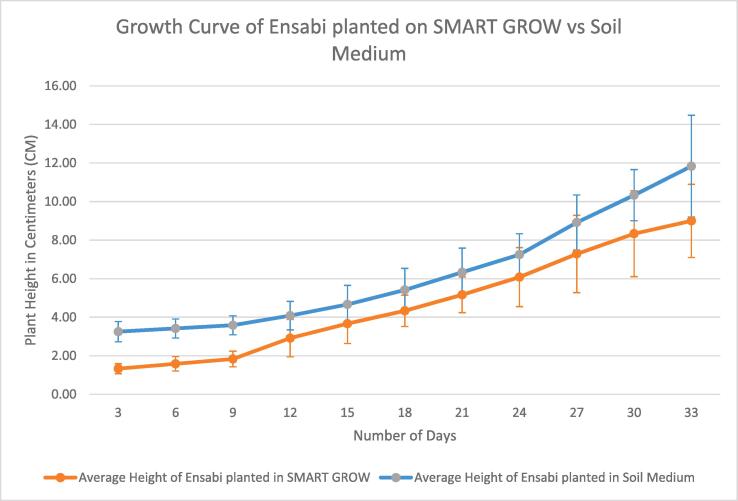
Growth curve of *Ensabi* planted on SMART GROW vs Soil Medium. Error bars indicate standard deviation.

**Fig. 22:**
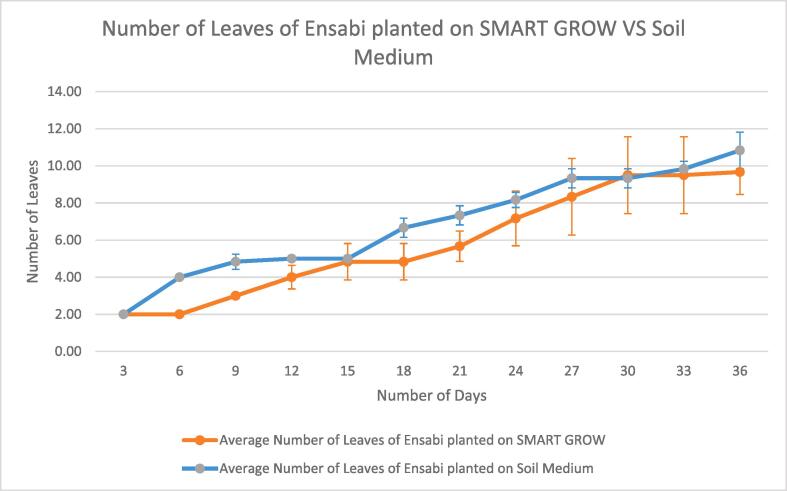
Graph Showing Average Number of Leaves of *Ensabi* planted on SMART GROW vs Soil Medium. Error bars indicate standard deviation.

Users were provided with SMART GROW hydroponic set and the mobile app. Hydroponic Nutrient AB was used in growing solution. Hydroponic Nutrients AB is a water soluble granular (SET A) and water-soluble powder (SET B) plant food containing N, Ca & Fe. It is a complete balanced fertilizer for hydroponics, indoor/outdoor potted plants, vegetables, and herbs. The plant species planted was *Brassica juncea (L.) Czern* var. *Ensabi* is a new local variety of mustard species, found only in Sabah and Sarawak, Malaysia. Users also cultivated *Ensabi* in conventional soil medium for observation. Users observed and documented the growth of *Ensabi*. Based on the results obtained from four weeks of planting till harvesting, the number of leaves and height are measured in three days intervals. (Fig. 21, Fig. 22). The average value was derived from data collected from 6 *Ensabi* plantlets at random grown with SMART GROW and Soil Medium. This shows that users can easily plant with SMART GROW (see Fig. 23a, Fig. 23b).

**Fig. 23a:**
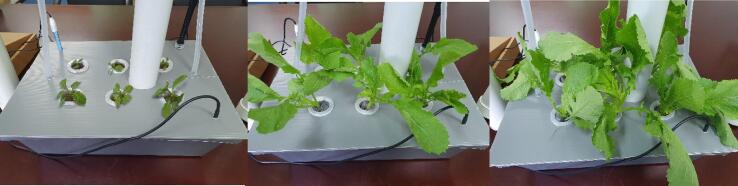
A series of picture showing *Ensabi* grown on SMART GROW at various stages till ready for harvesting.

**Fig. 23b:**
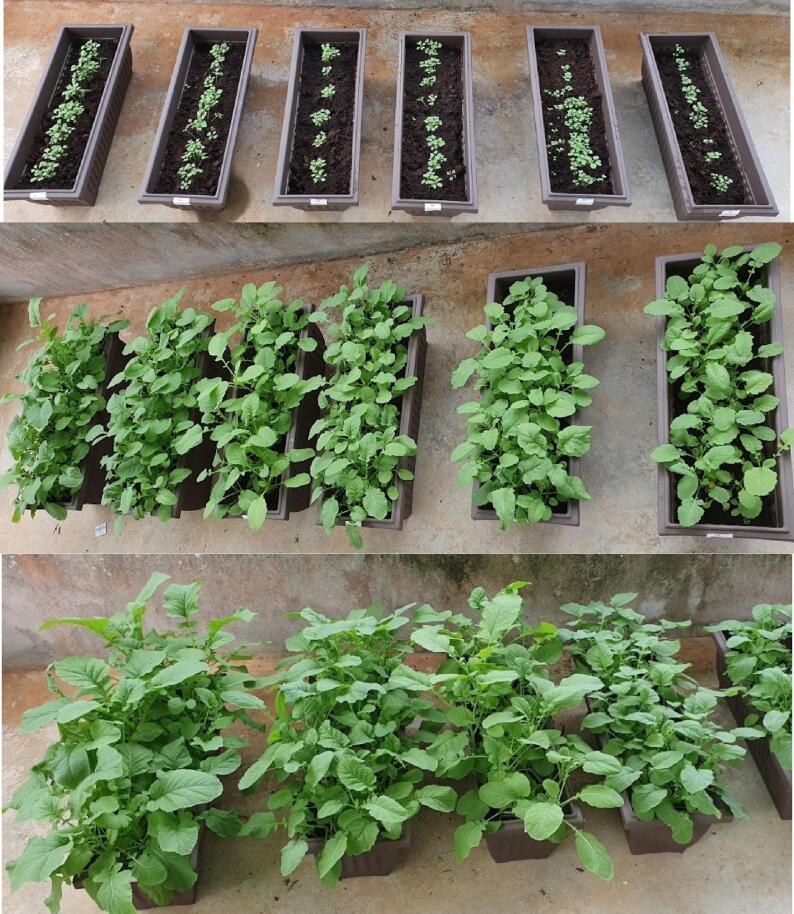
A series of picture showing *Ensabi* grown on soil medium at various stages till ready for harvesting.

## Ethics statements

No ethical approval is needed.

## Declaration of competing interest

The authors declare that they have no known competing financial interests or personal relationships that could have appeared to influence the work reported in this paper.
